# Cultural adaptation and preliminary validation of the Flexibility Scale for Spain

**DOI:** 10.3389/fpsyt.2024.1443078

**Published:** 2024-10-09

**Authors:** Ekaine Rodríguez-Armendariz, Valentina Petrolini, Isabel Martín-González, Maria Juncal-Ruiz, Agustín Vicente

**Affiliations:** ^1^ Department of Linguistics and Basque Studies, University of the Basque Country (UPV/EHU), Vitoria-Gasteiz, Spain; ^2^ Department of Psychiatry (Child and Adolescent Mental Health Unit), Sierrallana Hospital, Torrelavega, Spain; ^3^ Department of Medicine and Psychiatry, Instituto de Investigación Marqués de Valdecilla (IDIVAL), University of Cantabria, Santander, Spain; ^4^ Ikerbasque-Basque Foundation for Science, Bilbao, Spain

**Keywords:** Flexibility Scale, cultural adaptation, validation, autism spectrum disorder, flexibility, Spain

## Abstract

**Background:**

Individuals exhibit varying degrees of flexibility depending on different characteristics, conditions, and diagnoses. The Flexibility Scale is a comprehensive informant-report measure used to assess multiple aspects of flexibility in autistic and non-autistic children and youth, with an emphasis on cognitive aspects. This tool has not been adapted nor validated for use in Spain.

**Methods:**

In this paper we aim to provide a culturally adapted and validated version of the Flexibility Scale for Spain. We detail the procedure followed by our research team to obtain a cultural adaptation of the scale for Spain, including forward and back translations, cognitive interviews, feedback from experts, and preliminary validation. Our participants in the validation process were 75 autistic and 39 typically developing children and adolescents between the ages of 6 and 17.

**Results:**

We reached a culturally adapted and preliminary validated version of the Flexibility Scale for Spain (Escala de Flexibilidad).

**Conclusions:**

We make the scale available to researchers and practitioners working in Spain. This contributes to laying the groundwork for further testing and adaptations in Spanish-speaking countries and communities worldwide.

## 1 Introduction

Flexibility, a key component of executive functions, is typically described as the ability to shift to different thoughts or actions depending on situational demands (1), or to adjust one’s thinking and behavior to changed demands or priorities (2). Flexibility may be differentially affected in a range of conditions. In the context of autism research, issues with flexibility are variously described in the context of diagnostic manuals – e.g., insistence on sameness, inflexible adherence to routines, ritualized patterns of behavior, highly restricted or fixed interests (3). Considerations about flexibility are also common in clinical practice, where professionals often discuss intolerance of uncertainty, literalism, and the need for a highly structured environment. Although flexibility tends to be treated as a unitary concept, it is likely to encompass several facets that are not clearly distinguished from one another or fully examined in their relation [see (4) for a discussion].

A variety of instruments are currently employed in research, intervention, and clinical practice to measure different aspects of flexibility in connection with executive functioning abilities [e.g., Behavioral Rating Scale of Executive Function - BRIEF - (5)]. Researchers also routinely assess cognitive flexibility through a variety of tasks, including the Wisconsin Card Sorting Task, the Unusual Uses Task, and the Intradimensional–Extradimensional Shift task [see (1)]. Most of these tests recruit several executive functioning components, including working memory, inhibition, and task-switching, but are generally thought to be sufficiently good proxies of cognitive flexibility. Other scales, such as the Behavior Flexibility Rating Scale - Revised – BFRS-R (6), focus primarily on behavioral components of flexibility and include items aimed at assessing the person’s reaction to changes in their environment – e.g., “a commonly used object is misplaced and cannot be found”; “an activity is interrupted before the person was able to finish the task” (BFRS-R, **Appendix 1**). The Flexibility Scale [FS henceforth, (7)] has been proposed as a more global informant-report measure to assess multiple aspects of flexibility in autistic and non-autistic children and youth (6-17 years old). While other scales tend to focus on sub-components of flexibility, FS targets flexibility as a multifaceted trait that includes cognitive as well as behavioral aspects. Given that issues with flexibility are often regarded as central to autism, an effective and multi-faceted assessment of the construct contributes to improving our understanding of autism, along with the specific challenges faced by autistic individuals in this domain.

The Flexibility Scale is currently available in its original English version. This paper focuses on the linguistic and cultural adaptation of the scale and its preliminary validation for Spain. The linguistic adaptation’s main goal is to translate the scale appropriately, such that the original communicative intention for each item of the scale is preserved. Once this is done, the scale is applied to a sample of autistic and neurotypical participants, with the purpose of calculating the statistical properties of the translated instrument, such as reliability and validity. The final product is a Spanish-language version of the Flexibility Scale (Escala de Flexibilidad), which is a suitable tool for research and clinical practice in Spain. The adaptation and validation of the scale for Spain also lays the groundwork for further linguistic and cultural adaptations in Spanish-speaking countries and communities worldwide. In what follows, we detail the methodology and procedure followed by the research team to obtain the linguistically and culturally adapted scale.

## 2 Materials and methods

### 2.1 Flexibility Scale

The Flexibility Scale (7) is a multidimensional informant-report measure to assess real-world flexibility skills in children and youth between 6 and 17 years old. The original scale was validated on a sample of 221 participants diagnosed with Autism Spectrum Disorder (ASD) and 57 typically developing participants, and is composed of 27 items grouped into five factors (Routines/rituals, Transitions/change, Special interests, Social flexibility, and Generativity). Participants in the development and preliminary validation of this cognitive flexibility measure were to be intended as the autistic individuals whose behavior was reported by the informant (i.e., parent or caregiver) who completed the Flexibility Scale.

Since its publication, the scale has been employed in a range of studies investigating correlations between cognitive and behavioral flexibility in young autistic people and a variety of constructs, including externalizing and internalizing behavior (8), social and communication skills (9), emotional dysregulation (10), schema-based memory formation (11), and sensory sensitivity and selective eating (12, 13). The scale was also featured in a comprehensive review on clinical tools targeting cognitive and behavioral flexibility across clinical and nonclinical populations (14). Recently, an adaptation of the Flexibility Scale for autistic adults as a self-report measure has also been published (15).

### 2.2 Cultural adaptation

#### 2.2.1 Research team

All research team members had extensive experience in autism research and were responsible for the analysis of the original scale, the selection of experts and participants, the communication with professionals and members of the target population, and statistical analysis. The research team members also availed themselves of a range of professionals throughout the cultural adaptation process. **Table 1** summarizes the role played by each professional involved in the study.

**Table 1:** Research team and professionals.

Phases	Members	Expertise	Tasks
1. Research team	4	1 Occupational Therapist, 1 Psychologist, 2 Philosophers (all had experience in autism research)	Selection of participants; Analysis of the original scale.
2. Forward translation team (independent translators)	2	1 Occupational Therapist and 1 Psychologist (both fluent in English and Spanish)	Translation of the original scale from English to Spanish.
3. Synthesis of the forward translations	1	1 Psychologist (fluent in English and Spanish)	Synthesis of the two Spanish translations.
4. Back translation	1	1 Linguist (fluent in English and Spanish)	Translation of the synthesized scale from Spanish to English.
5. Equivalence team	4	Research Team (see 1.)	Preparation of the preliminary version of the scale translated into Spanish.
6. Experts panel team (independent, not part of the research team)	2	1 Occupational Therapist and 1 Psychologist (both experts in autism intervention and assessment)	Review of the preliminary version of the scale in Spanish
7. Cognitive interview team	4	Research Team (see 1.)	3 relatives and 3 caregivers interviewed to obtain feedback on the comprehension of the translated items.
8. Correspondence with first author of the original scale	2	Research Team (see 1.)	Query about items that raised comprehension or response difficulties during the cognitive interviews.
9. Linguistic expert (independent, not part of the research team)	1	Linguist	Review of the items to adapt the scale to Spanish from a linguistic viewpoint (e.g., grammar, semantics).
10. Final review team	4	Research Team (see 1.)	Final revision of the items and adjustment to the suggestions collected in steps 8. and 9. Preparation of the final version of the adapted scale.

#### 2.2.2 Procedure

**Figure 1:**
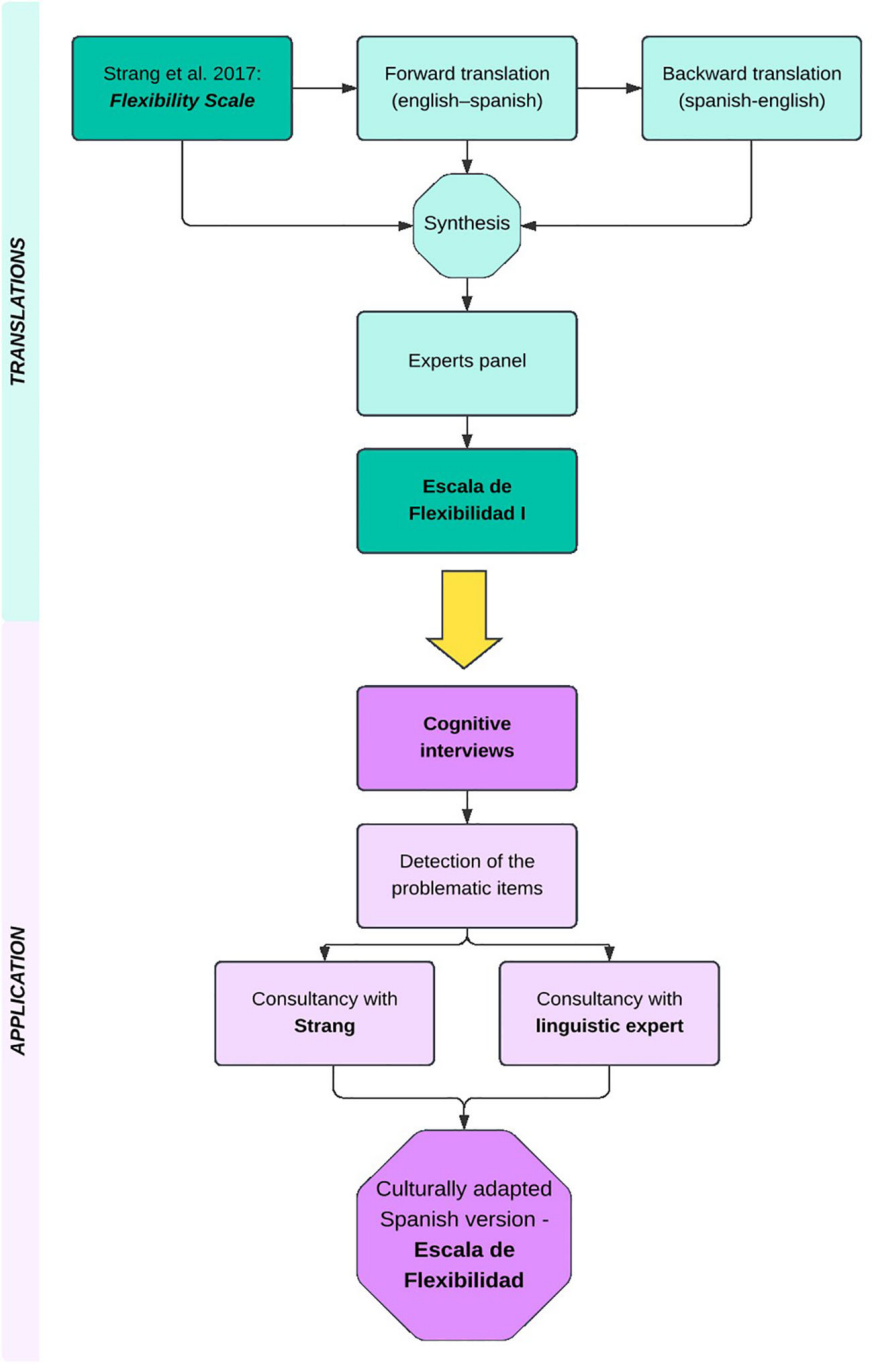
Diagram representing the steps followed for the linguistic and cultural adaptation of the Flexibility Scale for Spain.

We followed several international guidelines for cultural adaptation, such as the ITC Guidelines for Translating and Adapting Tests (16); Ramada-Rodilla et al. (17); Borsa et al. (18); Muñiz et al. (19); Sousa and Rojjanasrirat (20). These recommended forward and back translation, a panel of experts, cognitive interviews, and linguistic expert review. To design the cognitive interviews we also relied on further guidelines and examples detailed by Benes (21) and Caicedo Cavagnis and Zalazar-Jaime (22). Finally, we looked at other instruments and scales that had been adapted to Spanish to obtain a clearer picture of the steps involved as well as of potential challenges (23–25). Some of the sources that we used were specifically focused on cultural adaptations of existing scales or instruments from English to Spanish (17, 19). The complete procedure is represented graphically in the following diagram (**Figure 1**). After the diagram, we offer more details on each step.

##### 2.2.2.1 Translations and Panel of experts

Two independent experts, an occupational therapist and a psychologist, both familiar with the notion of flexibility in autism in a clinical setting, as well as native speakers of Spanish and fluent in English, translated all the items of the scale.

A third independent expert in autism, a psychologist also fluent in English and Spanish, carried out the task of synthesizing both translations. This was done either by selecting the more appropriate translation between the two direct translations available, or by offering a novel one focused on the equivalence between the original version and the direct translations.

Another independent translator, a linguist, translated the synthetic version of the scale back into English. The translator was a native speaker of English, fluent in Spanish and familiar with Spanish culture, but not familiar with research in autism and flexibility. The back translation was performed as a further quality check, as recommended by some of the resources consulted (23).

The research team then performed a review of the back translation, the original instrument, and the synthetic direct translation, looking for further inconsistencies and mistakes. The final output was a preliminary draft of the Spanish version of the scale.

All documents produced in the previous steps were sent to two experts in autism – i.e., one occupational therapist and one psychologist (these were different from the experts making up the forward translation team). Their purpose was to assess the equivalence between the original scale and the preliminary draft and appraise the scale’s adequacy as an assessment measure. All the issues raised by the experts were taken into account at later stages.

##### 2.2.2.2 Application: cognitive interviews and experts feedback

The goal of this phase was to test the comprehensibility and adequacy of the items for the target population – i.e., family members or caregivers of autistic children or young adults [as recommended by the (16)].

Six participants were selected for this phase – i.e., 3 parents and 3 caregivers who lived and/or worked in close contact with autistic children and young adults. All the participants were native speakers of Spanish. The children or service users had to be between 4 and 20 years old and received a diagnosis of Autism Spectrum Disorder.^1^ Through a short interview, members of the research team asked the participants about the comprehensibility of each item, their ability to rephrase it, and whether they were able to offer concrete examples that would support their answer. All the interviews were audio recorded, and the research team collected feedback in terms of understanding and paraphrasing, as well as suggestions to improve comprehension.

Items that raised issues in the cognitive interviews were then discussed with the first author of the original Flexibility Scale (John F. Strang). Through this step, the research team also obtained information about the original intention behind each item, thereby managing to adapt the translation in cases where it had to be significantly different from the original (e.g., idiomatic expressions). The author of the original scale supplemented the information related to some items to ensure that meaning was preserved during adaptation. Following the cognitive interviews and the exchange with the author of the original version, the research team made further linguistic adjustments to enhance comprehensibility and to modify items according to the feedback received.

The final adapted version, along with all the documents produced in the previous phases, was sent to an independent researcher in linguistics for a final review. Besides checking the items that raised issues in the cognitive interviews, the expert also provided feedback about the solutions that the research team suggested after corresponding with the author of the original scale. The objective was to obtain a version that was closest to the original scale from a linguistic point of view (e.g., grammar, semantics). After reviewing the suggestions of the expert in linguistics, the research team approved the final version of the scale.

### 2.3 Preliminary validation

The main purpose of validation was to assess whether the internal consistency of the instrument was maintained after its translation, i.e., whether it was still a reliable tool to test autistic participants. We also calculated whether the translated scale was able to discriminate between typically developing (TD) and ASD participants, i.e., discriminant validity, and its degree of correlation with the ADOS-2 scores and subscores [see (7)]. This last measure would give information about the utility of using a combination of both tools (ADOS-2 and Flexibility Scale) to diagnose autism. A strong correlation between them would thus signal that both instruments test similar underlying constructs. However, if correlation is very low or absent, it would mean that they tap into different aspects of autism. This information is important to understand the purposes of both instruments, and can be used to make decisions on when to privilege one or the other – or when to use them jointly – as assessment and diagnostic tools. We planned this strategy for preliminary validation of culturally adapted scales following Sousa and Rojjanasrirat (20) and Ramada-Rodilla et al. (17) recommendations, as well as Strang et al.’s own validation (2017), in which they correlate the Flexibility Scale with a sizable amount of other instruments such as BRIEF and the Wechsler scales, as well as ADOS-2, to understand its scope as a diagnostic tool. Our validation should be considered to be preliminary data due to the reduced size of the ASD sample (N = 75).

#### 2.3.1 Participants

Participants were 75 autistic children and adolescents (56 males and 19 females) between the ages of 6 and 17 (M = 11.91 SD = 2.83), and 39 typically developing children (23 males and 16 females) (M = 8.23, SD = 3.09). The TD children were included in the study to investigate the capacity of the adapted version of the FS scale to discriminate between autistic and non-autistic individuals. Only children who did not exhibit genetic, neurological, cognitive or linguistic conditions were included in the TD group. Participants within the autistic group (hereafter ASD sample) received a clinical diagnosis of ASD (n = 15) or were assessed in our lab through the ADOS-2 and crossed the clinically relevant threshold for autism (n = 60). Only children with verbal fluency were included in the autistic group, as required by the FS scale.

#### 2.3.2 ADOS-2

The ADOS-2 (26) is the revised version of the ADOS (27). The ADOS is a semi-structured, standardized and observational assessment of reciprocal social interaction, communication, repetitive behaviors, and stereotyped interest patterns for individuals suspected of having autism spectrum disorders. There are five different modules of the ADOS-2, each designed for a different developmental/communication level for ages between 12 months and adulthood. Most participants of the ASD sample in this study were assessed with the ADOS-2 Module 3 (N = 54) or the ADOS-2 Module 4 (N = 6), designed for participants with fluent language.

#### 2.3.3 Procedure

Participants were evaluated by the ADOS-2 as a part of a research protocol at the Language in Neurodiversity Lab (Lindy Lab) at the University of the Basque Country (UPV/EHU), or as part of the clinical assessment for autism in a local hospital’s child psychiatry unit. The participants with an Autism Spectrum or Autism classification according to the ADOS-2 were included in the present study. Participants with a clinical diagnosis of Autism Spectrum Disorder and fluent language were also included in the study.

All parents or legal representatives of the participants were contacted by email or telephone to invite them to participate in the study. Those who accepted the invitation were provided with an informed consent form. Once the consent form was signed, the parents were sent a link to a survey that they could complete electronically. All participants who completed the survey in full were included in the study. Ethical approval for this study was issued by University of the Basque Country’s (UPV/EHU) Ethics Committee for research with human beings (CEISH), code M10_2019_205.

## 3 Results

### 3.1 Cultural adaptation

#### 3.1.1 Translations

After reviewing the synthetic translation alongside the back translation, the research team detected some items in which conceptual or linguistic equivalence had not been fully accomplished. For example, item 12 (“insists on sameness”) did not have an obvious translation in Spanish and was translated literally as “insistencia en lo mismo”. Given the difficulty in capturing the nuances of this expression, widely employed in autism research, the research team suggested a solution (“insiste en que las cosas sucedan siempre de la misma manera”) which was retrieved from Spanish sources specialized in autism (e.g., *Confederación Autismo España*). The research team’s proposal was added to the draft version of the translation. The table was then sent to the panel of experts, who were asked to comment on linguistic as well as conceptual equivalence of each item and to offer further feedback. To exemplify the process, in **Table 2** we illustrate the cases of items 6 and 10.

**Table 2:** Examples of item modification throughout Translations.

Item #	6	10
**Original Item**	Difficulty with change of routine/schedule.	Rigid about rules; legalistic.
**Forward translation 1**	Tiene dificultad con los cambios en rutinas/horarios.	Es rígido con las normas, es decir, le gusta seguirlas sin excepciones.
**Forward translation 2**	Dificultades cuando hay cambios de rutina u horarios.	Es rígido con las reglas; legalista.
**Synthesis**	Tiene dificultad con los cambios en rutinas/horarios.	Es rígido con las normas, es decir, le gusta seguirlas sin excepciones.
**Back translation**	S/he has difficulty with changes in routine/timetable.	S/he is rigid in following rules. That is to say, s/he likes to follow them without exception.
**Preliminary Spanish version**	Tiene dificultad con los cambios en rutinas/horarios.	Es rígido con las normas, es decir, le gusta que se sigan sin excepciones.
**Evaluation of equivalence**	Yes	No
**Experts panel**	Agreement *(item approved)*	No agreement *(item left pending to collect more feedback through the cognitive interviews)*

#### 3.1.2 Application

##### 3.1.2.1 Cognitive interviews

No relevant cultural issues emerged during the cognitive interviews. However, the interviews underscored several linguistic and comprehension issues that had to be addressed. Out of the 27 items listed in the scale, 19 were easily understandable and did not present comprehension issues (items 1, 2, 3, 6, 7, 8, 11, 12, 13, 14, 15, 16, 17, 18, 20, 21, 22, 23, 27), 1 item created comprehension difficulties for one participant (item 25), 4 items created comprehension difficulties for two participants (items 9, 10, 24, 26), and 3 items created comprehension difficulties for three or more participants (items 4, 5, 19). The eight items identified as problematic by one or more participants were included in a further revision step. The difficulties of understanding and interpretation were mainly due to the understanding and use of certain terms and to the use of idiomatic expressions in the original version. For example, item 5 (“Is a ‘good sport’”, translated as “Se toma las cosas con deportividad”) presented comprehension issues as the broader implications of this idiomatic expression were not clear, and most participants thought it was exclusively referring to situations involving sports and games. Another example is item 9 (“Builds on ideas of others in conversations”, translated as “Conversa basándose en las ideas de otros”) where problems with the understanding of “*basarse en las ideas*” which is a literal translation of “*builds on ideas*” were identified.

##### 3.1.2.2 Correspondence with first author of the original scale

The issues identified in the cognitive interviews were also discussed with the first author of the original scale to solve items related to difficulties of understanding or interpretation (items: 4, 5, 9, 10, 19, 24, 25, 26). After this correspondence, the research team was able to achieve a version of some items that reflected more closely the intentions of the original authors. One example concerns the items related to creativity, which included some idiomatic expressions such as item 25, *“Thinks outside of the box”* (initially translated as *“Tiene facilidad para pensar fuera de lo establecido”*). After the correspondence, we opted for a paraphrased but more straightforward version in Spanish: *“Piensa en las cosas de manera inusual/no habitual”.*

##### 3.1.2.3 Linguistic review

The research team provided the expert with a spreadsheet listing the 27 items of the scale and divided into three groups. “Non-problematic items” were the ones that did not raise specific issues in the cognitive interviews (items 1, 2, 3, 6, 7, 8, 11, 12, 13, 14, 15, 16, 17, 18, 20, 21, 22, 23, 27). “Solved after consulting the original author” items were a group of items that raised issues during the cognitive interviews, but that the research team had solved following the recommendations of the author of the original scale (items 4, 9, 24, 25, 26). “Problematic items” were the ones which raised issues during the cognitive interviews and whose difficulties had not been fully solved after consulting the author of the original scale (items 5, 10, 19). The linguistic review brought about several modifications in order to adapt the scale to semantic and grammatical norms in Spanish. Some of the proposed modifications involved nuances in the meaning of specific words, such as in item 23: *“Gets upset when losing a game” > “Se enfada cuando pierde en un juego” > “Se molesta cuando pierde en un juego”*. Others included a rephrasing of idiomatic expressions to convey their meaning and implications more accurately. For example, the proposed changes in the aforementioned item 5 (*“Is a good sport” > “Toma las cosas con deportividad” > “Encaja bien las contrariedades en sus interacciones sociales”*) allowed the research team to approximate the broader meaning of the original expression.

**Appendix 1** (in **Supplementary Material**) shows the linguistic and culturally adapted version of the Flexibility Scale (Escala de Flexibilidad) for Spain.

### 3.2 Preliminary validation

#### 3.2.1 Data analysis: reliability and validity of the culturally adapted FS

Statistical analyses to assess internal reliability and discriminant validity were conducted using R Statistical Software (28). Internal consistency was evaluated through Cronbach’s α with the *psych* library (29), with values between 0.70 and 0.79 being considered acceptable and values >.80 being considered good (30).

Discriminant validity, on the other hand, was calculated by comparing the mean results in the scale obtained by both the TD and the ASD group. Marginal means were computed for both samples using the *emmeans* package (31). Later, the FS total score was calculated for a sub-group of the original sample, comprising the participants who had been assessed through the M3 of the ADOS-2 (N=54). For this sub-group, the total FS score was analyzed along with the ADOS-2 severity measures, Global Total Score, Social Affectation Score and Restrictive and Repetitive Behaviors Score. These analyses were conducted using linear regressions and *p*-values <0.05 were considered significant. Normality assumptions were visually checked in the model residuals.

Reliability was evaluated through the Internal Consistency of the FS in the two samples (by calculating Cronbach’s α). Internal reliability was acceptable for the control sample (α = 0.72) and good for the ASD sample (α = 0.85). **Table 3** shows the changes in Cronbach’s alpha value for the TD and the ASD sample if an item is deleted. There was no significant increase or decrease in reliability values after an item was deleted in both samples, so all items were retained. Nevertheless, when testing for internal reliability of the subscales that make up the FS, as described in the original article (7) the resulting reliability scores were notably below the acceptance threshold (α = 0.70). Internal reliability was slightly higher in the ASD sample (**Table 4**).

**Table 3:** Flexibility Scale reliability scores (Cronbach’s α) for TD and ASD samples if Item deleted.

Item	Cronbach’s Alpha if Item is dropped	
	TD	ASD
**1**	0.751	0.851
**2**	0.716	0.851
**3**	0.715	0.849
**4**	0.711	0.842
**5**	0.717	0.847
**6**	0.723	0.838
**7**	0.721	0.845
**8**	0.728	0.846
**9**	0.719	0.849
**10**	0.722	0.841
**11**	0.698	0.847
**12**	0.693	0.835
**13**	0.696	0.844
**14**	0.696	0.847
**15**	0.726	0.846
**16**	0.698	0.843
**17**	0.693	0.845
**18**	0.701	0.843
**19**	0.707	0.848
**20**	0.697	0.836
**21**	0.704	0.838
**22**	0.712	0.845
**23**	0.720	0.845
**24**	0.743	0.856
**25**	0.761	0.861
**26**	0.727	0.856
**27**	0.679	0.841

**Table 4:** Flexibility Scale and sub-scales reliability scores (Cronbach’s α) for TD and ASD samples.

	TD	ASD
**Total**	0.723	0.851
**Routines/rituals**	0.478	0.634
**Transitions/change**	0.121	0.526
**Special interests**	0.051	0.662
**Social flexibility**	0.335	0.545
**Generativity**	0.646	0.781

**Figure 2:**
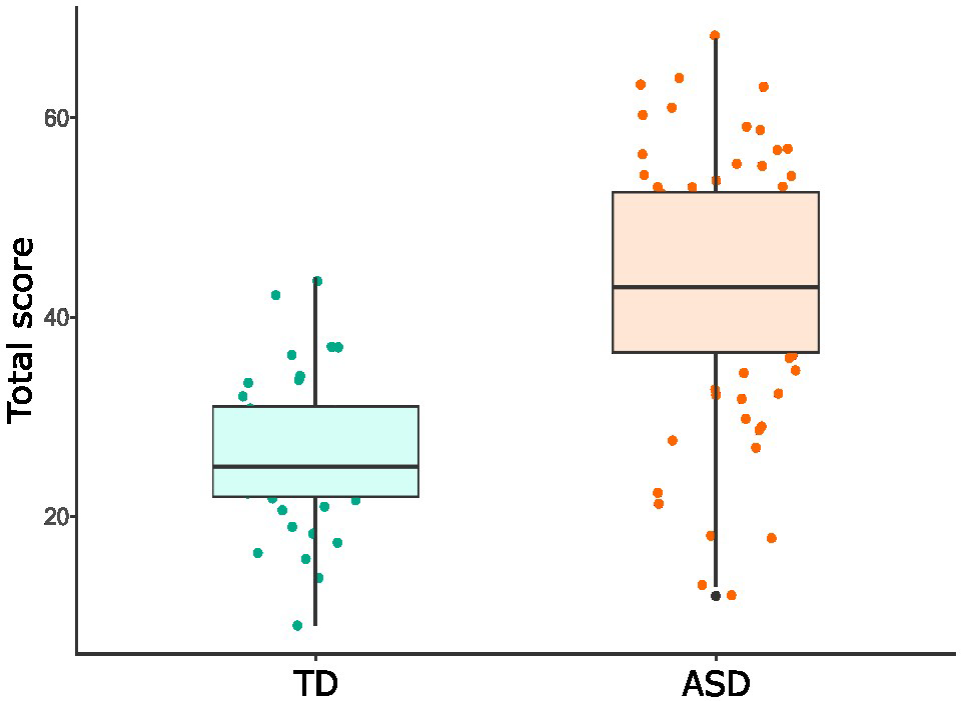
Boxplot representing the Flexibility Scale total score for TD and ASD samples. Points depict each participant's total score. Lower and upper hinges correspond to the 25th and 75th percentiles, whiskers show 95% confidence intervals and the horizontal solid line represents the median.

The total score of the FS exhibited discriminant validity as it reliably differentiated between typically developing children [Mean (CI) = 26.3 (22.9 - 29.7)] and autistic children [Mean (CI) = 43.2 (40.7 - 45.6)]. F value = 62.28 and p < 0.001 (**Figure 2**).

Analyzing the FS total score into the ASD sample for the participants that completed the M3 on the ADOS-2 showed a lack of relationship between the FS and the ADOS-2 M3 for: severity measures (F value = 0.62 and *p* = 0.43), Global Total Score (F value = 0.75 and *p* = 0.38), Social Affectation Score (F value = 1.42 and *p* = 0.24) and Restrictive and Repetitive Behaviors Score (F value = 0.88 and *p* = 0.35).

## 4 Discussion

### 4.1 Linguistic and cultural adaptation

This study reports on the linguistic and cultural adaptation as well as validation of the Flexibility Scale for Spain (7). Our final version of the scale (see **Appendix 1** in **Supplementary Material**) exhibits conceptual and semantic equivalence to the original English version. The use of independent translators allowed the research team to obtain substantial feedback on the best ways to convey each item, while at the same time minimizing any bias due to previous familiarity with the original scale.

Yet, translations are rarely sufficient to ensure suitability (16). In this study, the research team availed itself of further resources: a third translator offered a synthetic version starting from the direct translations available, and the panel of experts assessed the adequacy of the synthetic translation. The latter step was not simply meant to resolve any discrepancies between the original instrument and the provisional translation, but also to make sure that the translated scale would work adequately as an assessment measure. This was done by taking into account the features of the target population, in terms of nationality or culture as well as in more specific ways – e.g., their features as families and caregivers of autistic children and young individuals. Finally, through the cognitive interviews, the research team collected a wide range of observations by a sample of the target population. After the interviews, the research team also corresponded with the original author by sending them proposed solutions, thereby trying to reach an agreement so that the core meaning of the item would be maintained while conveying the appropriate message to the target population.

The methodology for cultural adaptation described in this report provides a model for other research groups who wish to develop cultural adaptations of existing scales in autism research. Assessment instruments specific to this target population have to be sufficiently technical to provide support for research and professional purposes while also being easily understandable to the informants. The translation phase aimed to preserve the technical quality of the scale by focusing on the intended content of the original items. The application phase aimed to collect more information by consulting with members of the community, who greatly contributed to making the instrument more adequate.

By making the adapted scale available to researchers we also facilitate the validation process that would eventually allow practitioners and researchers to incorporate the scale in their everyday activity. Given that the Flexibility Scale is one of the few existing measures that targets rigidity as a multifaceted trait, comprising both cognitive and behavioral components, our adapted version allows professionals working in Spain to include it in studies aimed at assessing real-world flexibility skills, as well as in studies interested in using inflexibility or any of its specific sub-dimensions as predictors or as grouping variables. This is particularly important given the gap between cognitive and behavioral flexibility measures that has been emphasized in the literature. While autism research has mostly approached the notion of flexibility through the executive function paradigm (32), the current measures of cognitive flexibility (e.g., the WCST) are often insufficient to capture the complexity of factors that affect behavioral flexibility, i.e., the ability to adapt to different contexts and tasks in real life situations.

### 4.2 Validation

As explained above, the adaptation of Strang et al.’s Flexibility Scale was administered to 75 caregivers of autistic children and to 39 caregivers of typically developing children. The total score of the adapted scale showed internal consistency and a discriminant capacity between typically developing children and autistic children, including the ones assessed through the ADOS-2 who obtained an Autism Spectrum or Autism classification and the ones who came to the lab with a previous diagnosis (F value = 62.28 and p < 0.001), thereby exhibiting discriminant validity. Comparison between groups shows significantly higher scores in the autistic than in the non-autistic group, as expected. Yet, possibly due to the relatively small sample, the adapted version of the scale did not exhibit construct validity for the FS subscales. Such a result shows that the FS is a valuable addition to existing diagnostic tools. The FS can be an ideal complementary tool to existing screening questionnaires and scales such as the BRIEF or the BFRS-R, as well as to the ADOS-2 diagnostic tool. While the ADOS-2 is arguably the most complete diagnostic tool existing today, it does not examine flexibility traits directly. Thus, the lack of relations between FS scores and different ADOS-2 scores is not surprising [similar results were obtained by (7)]. This part of the current results has special significance because it suggests that the FS is a valuable complementary tool to ADOS-2 observations.

### 4.3 Limitations and conclusion

The first limitation concerns the applicability of the adapted scale to Spanish-speaking populations worldwide, given that cognitive interviews only recruited participants from different Spanish regions. The primary goal of this work is to make the scale accessible to Spanish-language researchers and practitioners working on flexibility in autism to allow for more efficient and widespread testing of this construct. Further studies will ensure that the scale is both understandable and appropriate to broader linguistic and cultural contexts.

Another limitation concerns the original scale, which was designed to test inflexibility patterns in the verbal population with a typical range of cognitive abilities. As mentioned above, we had initially decided to widen the age range of participants in the cognitive interviews to assess whether the scale would be applicable to a broader population also in terms of cognitive and linguistic abilities. Yet, we found that items regarding conversations (i.e., item 16 “Special interests interfere with conversation”), or creativity (i.e., item 24 “Easily generates new ideas, can brainstorm”) are scarcely applicable in the case of participants with a wider range of verbal and intellectual abilities. This opens up the possibility that autistic individuals with different abilities may display different flexibility profiles, which would be hardly captured by this instrument.

The results of this study provide evidence that the Spanish-language version of the Flexibility Scale is a suitable tool for use in clinical practice and research in Spain. They also lay the groundwork for further application and adaptation of the scale in Spanish-speaking countries and communities worldwide. A more widespread use of the FS will constitute a valuable addition to existing diagnostic and assessment tools for autism, a context where challenges with flexibility are often observed but rarely measured directly.

## Data Availability

The raw data supporting the conclusions of this article will be made available by the authors, without undue reservation.

## Footnotes

1 Although the original scale was validated for participants between 6 and 17 years of age, we decided to conduct our cognitive interviews with a wider age range in order to include participants exhibiting different linguistic abilities (e.g., older participants with concurrent linguistic disabilities). We further discuss this aspect in the Limitations section.
