# Supplementary material for: Cultural adaptation and preliminary validation of the Flexibility Scale for Spain
Source: Front Psychiatry. 2024 Oct 9;15:1443078. doi: 10.3389/fpsyt.2024.1443078 (PMC11496269; doi:10.3389/fpsyt.2024.1443078)
Supplement: Supplementary file 1 [file DataSheet1.pdf]

Supplementary Material

Cultural adaptation and preliminary validation of the Flexibility Scale for Spain

Ekaine Rodríguez-Armendariz*, Valentina Petrolini, Isabel Martín-González, Maria Juncal-Ruiz, Agustín Vicente

*** Correspondence:** Ekaine Rodríguez-Armendariz: ekaine.rodriguez@ehu.eus

Appendix 1. Culturally adapted and preliminary validated scale for Spain.

ESCALA DE FLEXIBILIDAD.

Adaptación de Strang et al. 2017 (*Flexibility Scale*).

**Tu nombre:**	**Fecha de hoy:**
**Nombre del niño/a:**	**Tu relación con el niño/a:**
**Fecha de nacimiento del niño/a:**	**Sexo del niño/a:**

Piensa sobre el comportamiento del niño/adolescente en las últimas dos semanas, y para cada pregunta, utiliza la siguiente escala para puntuar su comportamiento.

#	Ítem	Nunca	Algunas veces	Muchas veces	Siempre
**1**	Hace algo especial a la hora de acostarse. Por ejemplo: tiene que ajustar bien la manta sobre la almohada antes de irse a la cama.	0	1	2	3
**2**	Hay algo especial que necesita hacer cuando lo/a llevan o lo/a recogen del colegio/de las extraescolares. Por ejemplo, por las mañanas, lo primero es colgar el abrigo en el perchero, luego beber un poco de agua y después darle los buenos días a la profesora.	0	1	2	3
**3**	Hay algo que tiene que hacer en un orden concreto (dejando de lado las rutinas a la hora de ir a dormir o para ir al colegio).	0	1	2	3

**4**	Necesita ir por un camino concreto a los sitios conocidos.	0	1	2	3
**5**	Encaja bien las contrariedades en sus interacciones sociales	0	1	2	3
**6**	Tiene dificultad con los cambios en rutinas/horarios.	0	1	2	3
**7**	Comparte sus juguetes y otras pertenencias.	0	1	2	3
**8**	Le interesan las aficiones e intereses de otras personas.	0	1	2	3
**9**	Conversa de manera recíproca, acerca de ideas que otros han comentado.	0	1	2	3
**10**	Es rígido con las normas sociales, es decir, quiere que se sigan sin excepciones	0	1	2	3
**11**	Perfeccionista, intolerante con los errores o las pequeñas desviaciones.	0	1	2	3
**12**	Insiste en que las cosas sucedan siempre de la misma manera.	0	1	2	3
**13**	Dibuja, habla, o escribe sobre los mismos temas repetidamente. Por ejemplo: habla de coches todo el tiempo.	0	1	2	3
**14**	Finge ser el mismo personaje habitualmente (por ejemplo: Batman).	0	1	2	3
**15**	Insiste en llevar consigo algún objeto.	0	1	2	3
**16**	Sus intereses especiales interfieren con el desarrollo de la conversación.	0	1	2	3
**17**	Es más sociable si se habla de sus intereses especiales.	0	1	2	3
**18**	Le gusta saber todo sobre un tema en concreto.	0	1	2	3

**19**	Disfruta categorizando la información (por ejemplo, organizando imágenes, haciendo listas, pensando en trayectos y horarios de transportes, etc.).	0	1	2	3
**20**	Se queja cuando se le pide que haga las cosas de manera diferente.	0	1	2	3
**21**	No consigue cambiar de manera de pensar o actuar, aunque se le pida que lo haga.	0	1	2	3
**22**	Tiene dificultad para respetar los turnos.	0	1	2	3
**23**	Se molesta cuando pierde en un juego.	0	1	2	3
**24**	Se le ocurren ideas nuevas fácilmente.	0	1	2	3
**25**	Tiene una forma de pensar original, fuera de lo común.	0	1	2	3
**26**	Soluciona los problemas de manera independiente y creativa.	0	1	2	3
**27**	Generalmente rígido/a o insistente.	0	1	2	3

For the original version of the Flexibility Scale, please see Strang, J.F., Anthony, L.G., Yerys, B.E., Hardy, K.K., Wallace, G.L., Armour, A.C., Dudley, K., & Kenworthy, L. (2017). The Flexibility Scale: development and preliminary validation of a cognitive flexibility measure in children with autism spectrum disorders. Journal of Autism and Development Disorders. doi: 10.1007/s10803-017-3152-y
